# ‘Meta-analysis of dry matter intake and neutral detergent fiber intake of hair sheep raised in tropical areas’

**DOI:** 10.1371/journal.pone.0244201

**Published:** 2020-12-22

**Authors:** Alessandra Pinto de Oliveira, Camila Soares Cunha, Elzânia Sales Pereira, Stefano Biffani, Ariosvaldo Nunes de Medeiros, Aderbal Marcos de Azevedo Silva, Marcos Inácio Marcondes

**Affiliations:** 1 Department of Animal Science, Universidade Federal do Ceará, Fortaleza, Ceará, Brazil; 2 Department of Animal Science, Universidade Federal de Viçosa, Viçosa, Minas Gerais, Brazil; 3 Istituto di Biologia e Biotecnologia Agraria, Consiglio Nazionale delle Ricerche, Lodi, Italia; 4 Department of Animal Science, Universidade Federal da Paraíba, Areia, Paraíba, Brazil; 5 Rural Health and Technology Center, Universidade Federal de Campina Grande, Patos, Paraíba, Brazil; University of Florida, UNITED STATES

## Abstract

Inadequate estimates of fiber and dry matter intake of sheep raised in tropical conditions may explain part of the inefficiency of those production systems. Therefore, we aimed to estimate dry matter intake (DMI) and neutral detergent fiber intake (NDFI) of hair sheep raised under tropical conditions. A meta-analysis of 61 independent performance experiments, comprising a total of 413 experimental units (treatment means or animals), was performed. Trials were conducted in tropical conditions, using hair sheep in growing and finishing phases and endowed with the following information: neutral detergent fiber (NDF) in diet, initial and final body weight (BW), average daily gain (ADG), DMI and NDFI of treatment means (51 studies) or individual data (10 studies). Data on organic matter and NDF digestibilities were collected to estimate D-value (Dv) and B-value (Bv) (20 and 33 studies, respectively). The equations obtained were: ${DMI}_{(g/day)}=50.5773_{\pm 71.0504}+1.4423_{\pm 0.1704}\times ADG+28.4406_{\pm 2.9697}\times BW;\;{DMI}_{\left(g/kgBW\right)}=42.1088_{\pm 4.7298}+0.05516_{\pm 0.009427}\times ADG-0.4402_{\pm 0.1865}\times BW;\;{NDFI}_{(g/day)}={-52.2187}_{\pm 47.7718}+1.3773_{\pm 0.2292}\times {NDF}_{\left(g/kgDM\right)}-0.0007_{\pm 0.0002}\times {NDF}_{\left(g/kgDM\right)}^{2}.$ DMI (g/kg BW) as a function of Dv (g/kg DM) revealed a quadratic relationship, whose point of maximum DMI (38.69 g/kg BW) was obtained at 634.1 g/kg DM Dv. On the other hand, DMI decreased linearly as Bv (g/kg DM) increased. In conclusion, equations to predict DMI from BW and ADG as well to predict NDFI from dietary NDF were fitted with great accuracy and are recommended for hair sheep raised in tropical regions. DMI values were, in general, greater than those reported by the NRC, AFRC and INRA systems, which may be a reflection of the sheep breeds used in this study. Using Dv and Bv concepts was satisfactory to describe an integrated mechanism between metabolic and bulking regulation of DMI in sheep.

## Introduction

Feed intake is one of the most important variables for nutrition and metabolism, since it defines the quantity of ingested nutrients, thus determining the animal’s response [1]. Voluntary feed intake, however, varies according to diet, animal and environmental characteristics [2]. For instance, feedstuffs with low digestibility frequently limit dry matter intake (DMI) due to distension of gastrointestinal tract, particularly the reticulorumen. On the other hand, DMI is supposed to be regulated by metabolic feedbacks when diets with greater digestibilities are fed to ruminants [3]. Nevertheless, such a concept does not assume that both approaches could be integrated to modulate DMI [4–7]. In this sense, a study on the voluntary intake regulation of cattle, considering dietary total tract digestibility and bulkiness, concluded that physical constraints and metabolic feedbacks work together in the regulation of voluntary intake [8]. The authors evaluated the apparently digested organic matter, defined as the D-value (Dv) [9, 10], and the undigested fiber, defined as the B-value (Bv), which are associated with DMI and dietary neutral detergent fiber (NDF) [8]. Regarding animal characteristics, innate preferences and aversions, as well as previous experiences and nutrient supply, may lead to different feeding behaviors [7, 11]. Lastly, it is known that high temperatures also play an important role in decreasing feed intake [12]. On the other hand, animals exposed to low temperatures tend to increase intake in response to greater passage rate, due to an increase in the intestinal motility [7]. Thus, the integration of these characteristics is fundamental in mathematical models aiming to estimate animals’ feed intake.

Traditional committees of nutritient requirements for sheep [13–15] have been used to predict DMI in tropical environments. However, as previously mentioned, it is known that animals exposed to environments with high temperatures exhibit a reduction in feed intake [12] or lower production efficiency. Thus, DMI models developed in temperate countries, whose genotypes, diets and climate differ from those found in tropical regions, should be applied with caution to animals raised in tropical regions. Currently, there is only one study available that has performed a meta-analysis to estimate the DMI of hair sheep raised in tropical regions [16]; however, it did not consider Dv and Bv and did not study neutral detergent fiber intake (NDFI).

Therefore, we aimed to develop models to predict DMI and NDFI of hair sheep raised in tropical regions of Brazil, using a meta-analysis approach. The hypotheses were: 1) DMI of hair sheep raised in tropical regions is lower than those reported by the traditional committees of nutritient requirements for sheep [13–15]; 2) Dv and Bv are valid concepts to understanding DMI regulation in sheep.

## Materials and methods

Approval by an ethics committee on the use of animals was not necessary in this study, since data were collected from previously published studies.

### Inclusion criteria

A meta-analysis of 61 independent studies (totaling 413 experimental units, S1 Table in S1 File) was carried out. Peer-reviewed publications were compiled from the on-line public databases (e.g. Web of Science, CAB abstracts and Science Direct) by several searches conducted in February 2019. The following keywords were used during searches: “dry matter intake”, “lambs”, “sheep”, and “sheep in tropical environment”. In addition, data from theses published in Brazil were added to the dataset. Authors were contacted by email whenever necessary and the reference list of each paper and thesis were searched for titles that contained the keywords previously listed. Most of the studies aimed to evaluate responses to dietary change and inclusion of feed additives. All studies were conducted under tropical conditions (see meteorological information at S2 Table in S1 File), were published from 2002 to 2019 in English or Portuguese using hair sheep in growing and finishing phases, and endowed with the following quantitative information: NDF in diet, initial and final body weight (BW), average daily gain (ADG), DMI and NDFI of treatment means (51 studies) or individual data (10 studies). Data extraction was made independently. When data provided allowed the estimation of final BW or ADG, the study was kept in the dataset. Data on organic matter and NDF digestibilities were also collected to estimate Dv and Bv (20 and 33 studies, respectively). As Bv represents the undigested fiber, it was used in equations modelling as a variable that characterize rumen filling. Thus, differences between smaller and bigger lambs’ intake would be observed as the rumen filling is related to animal BW and rumen capacity. The description of the variables used in the meta-analysis is presented in Table 1.

**Table 1:** Descriptive statistics of the variables used in the meta-analysis to develop prediction equations of Dry Matter Intake (DMI) and Neutral Detergent Fiber Intake (NDFI) of hair sheep raised in the tropics.

Variable	n*	Minimum	Maximum	Mean	SD^†^
Forage, % TMR	397	0.00	80	48.91	15.23
Concentrate, %TMR	397	20.00	100	51.09	15.23
OM, %DM	186	80.69	96.20	91.18	2.81
CP, %DM	403	8.91	21.46	16.02	1.99
NDF, %DM	411	19.97	70.24	40.63	11.27
DMI, g/day	413	207.00	1700.00	971.23	240.84
DMI, %BW	413	7.42	79.90	39.19	8.45
NDFI, g/day	411	137.79	812.28	372.01	128.77
DOM, %	186	35.50	85.52	69.56	0.09
DNDF, %	226	13.10	80.92	47.80	0.14
D-value, g/kg DM	174	304.20	813.55	638.83	81.38
B-value, g/ kg DM	226	27.40	451.03	193.09	64.79
Average body weight, kg	389	13.25	34.63	24.69	4.21
Average daily gain, g/day	389	32.69	358.00	171.79	65.54

*n = number of experimental units (treatments or animals).

^†^SD = standard deviation.

TMR = total mixed ration; OM = organic matter; DM = dry matter; CP = crude protein; NDF = neutral detergent fiber; BW = body weight; DOM = organic matter digestibility; DNDF = neutral detergent fiber digestibility.

### Variables estimation

Equations used to estimate Dv and Bv were: 1) Dv = OM × D_OM_, where OM is the OM content of diet and D_OM_ is the OM total tract apparent digestibility (g/kg); and 2) Bv = NDF × (1 –D_NDF_), where D_NDF_ is the total tract apparent digestibility of NDF [8]. Organic matter and NDF contents as well D_OM_ and D_NDF_ values were collected from the papers used in the meta-analysis.

Mathematical models used to estimate the DMI and NDFI (expressed in g/day and g/kg BW) included the variables BW, ADG, NDF, Dv and Bv, as presented in Eqs 1–7. When the quadratic component was not significant, it was removed from the model (PROC MIXED SAS). Only data of animals fed above maintenance were used.

$${DMI}_{(g/day)}=\beta_{0}+\beta_{1}\times ADG+\beta_{2}\times {ADG}^{2}+\beta_{3}\times BW+\beta_{4}\times {BW}^{2}$$ (1)

$${DMI}_{\left(g/kgBW\right)}=\beta_{0}+\beta_{1}\times ADG+\beta_{2}\times {ADG}^{2}+\beta_{3}\times BW+\beta_{4}\times {BW}^{2}$$ (2)

$${DMI}_{(g/kgBW)}=\beta_{0}+\beta_{1}\times Dv+\beta_{2}\times {Dv}^{2}$$ (3)

$${DMI}_{(g/kgBW)}=\beta_{0}+\beta_{1}\times Bv+\beta_{2}\times {Bv}^{2}$$ (4)

$${DMI}_{(g/day)}=\beta_{0}+\beta_{1}\times NDF+\beta_{2}\times {NDF}^{2}$$ (5)

$${NDFI}_{(g/day)}=\beta_{0}+\beta_{1}\times NDF+\beta_{2}\times {NDF}^{2}$$ (6)

$${NDFI}_{(g/kgBW)}=\beta_{0}+\beta_{1}\times BW+\beta_{2}\times {BW}^{2}$$ (7)

where DMI = dry matter intake; NDFI = neutral detergent fiber intake; ADG = average daily gain (g/day); BW = body weight (kg); Dv = D-value (g/kg DM); Bv = B-value (g/kg DM); NDF = dietary neutral detergent fiber content (% DM); and *β*_*0*_, *β*_*1*_, *β*_*2*_, *β*_*3*_ and *β*_*4*_ = coefficients of the regression equations.

### Statistical analysis

In this meta-analysis, a random coefficients model was used [17], considering study as a random effect and including the possibility of covariance between the slope and the intercept. Data was weighted based on the number of replications [18] because individual data were accessed in some studies and because standard error was not available in some published studies. The effect of sex was tested in all model parameters, while breed was considered a random effect in the models, since Brazilian sheep breed composition is not accurate. Seventeen types of variance–covariance structures were tested and the Akaike’s Information Criteria (AIC) used to define the best fit. The covariance parameter was considered nonzero when the P-value was lower than 0.10. Individual outliers were removed when the Studentized residuals were greater than 2 or less than –2. When the Cook's distance [19] was greater than 1, the study was removed from the database in that particular analysis.

Models were tested for linear and quadratic effects (Eqs 1–7). To generate the tested models a backward elimination procedure was used, in which parameters from the initial equation that had P > 0.05 were removed. The initial model was complete (intercept, linear, and quadratic terms of the independent variable and its interactions). In the backward procedure, one term was removed from the initial equation at each step. Parameter exclusion was performed starting with the highest order, so when a term of higher order was significant (i.e., quadratic), the linear term was preserved in the model, even if it was not significant. After every round in which a parameter had been removed from the model, the analysis was restarted and the previously removed studies returned to the database. When the variable “sex” was not significant it was added to the model as a random effect. The significance levels assumed for fixed and random effects were 0.05 and 0.20, respectively. All attempts to fit Eq 5 resulted in low goodness of fit; thus, we specified a quadratic broken-line relationship to fit DMI as a function of NDFI [20]. We used the least-squares mean method to determine the point with maximum DMI in Eq 5. All statistical procedures were performed using the PROC MIXED of the statistical analysis software SAS version 9.2 (SAS Inst. Inc., Cary, NC), with the exception of Eq 5, which was fitted using PROC NLMIXED.

The cross-validation technique was used to evaluate the estimated models with 2,000 random replications [21, 22]. In this analysis, the database was randomly split in two groups: one group was used to fit the model and the other group to test the statistically estimated models [21]. For each simulation, models’ estimations were done using only the first group and adequacy of the models was estimated only from the other group [22]. These equations were estimated using the same variables of the selected equations. Adequacy statistics described were based on the mean value obtained from the 2,000 simulations. Then, the mean square error of prediction (MSEP), concordance correlation coefficient (CCC), and R^2^ were computed [21]. Three main sources of variation were considered in the MSEP decomposition [21, 23]: 1) mean bias, which represents a central tendency of deviation; 2) systematic bias, which is the deviation of the slope from 1; and 3) random error, consisting of the variation that is not explained by the regression. The CCC was decomposed into correlation coefficient estimate (ρ), which estimates model precision, and bias correction factor (Cb), which indicates accuracy [24]; thus, model accuracy and precision were accessed simultaneously. The values of CCC, ρ and Cb range from 0 to 1, in which precise and/or accurate models present values close to 1 [21, 24]. The R^2^ was computed as ρ^2^.

## Results

### Search results

**Fig 1:**
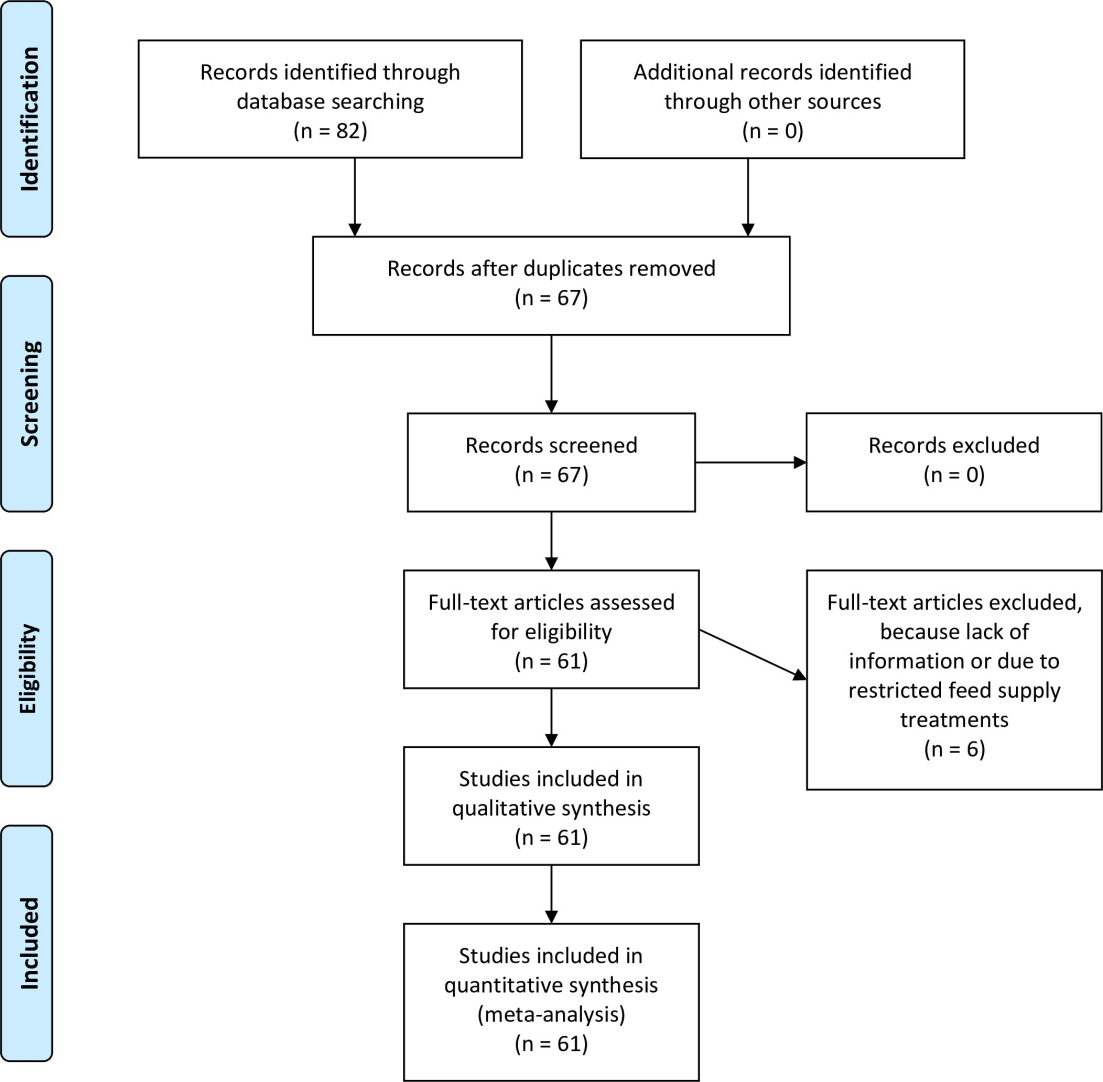
Literature retrieval flow chart.

The search in this meta-analysis was very limited because we used only experiments performed in tropical areas. Eighty-two papers met the search criteria and 13 were excluded due to duplication (Fig 1). From these 67 published papers and theses, six were removed due to the lack of necessary information or due to restricted feed supply treatments. The final data base consisted of 61 peer-reviewed articles or theses from 2002 to 2019.

### Dry matter intake

**Fig 2:**
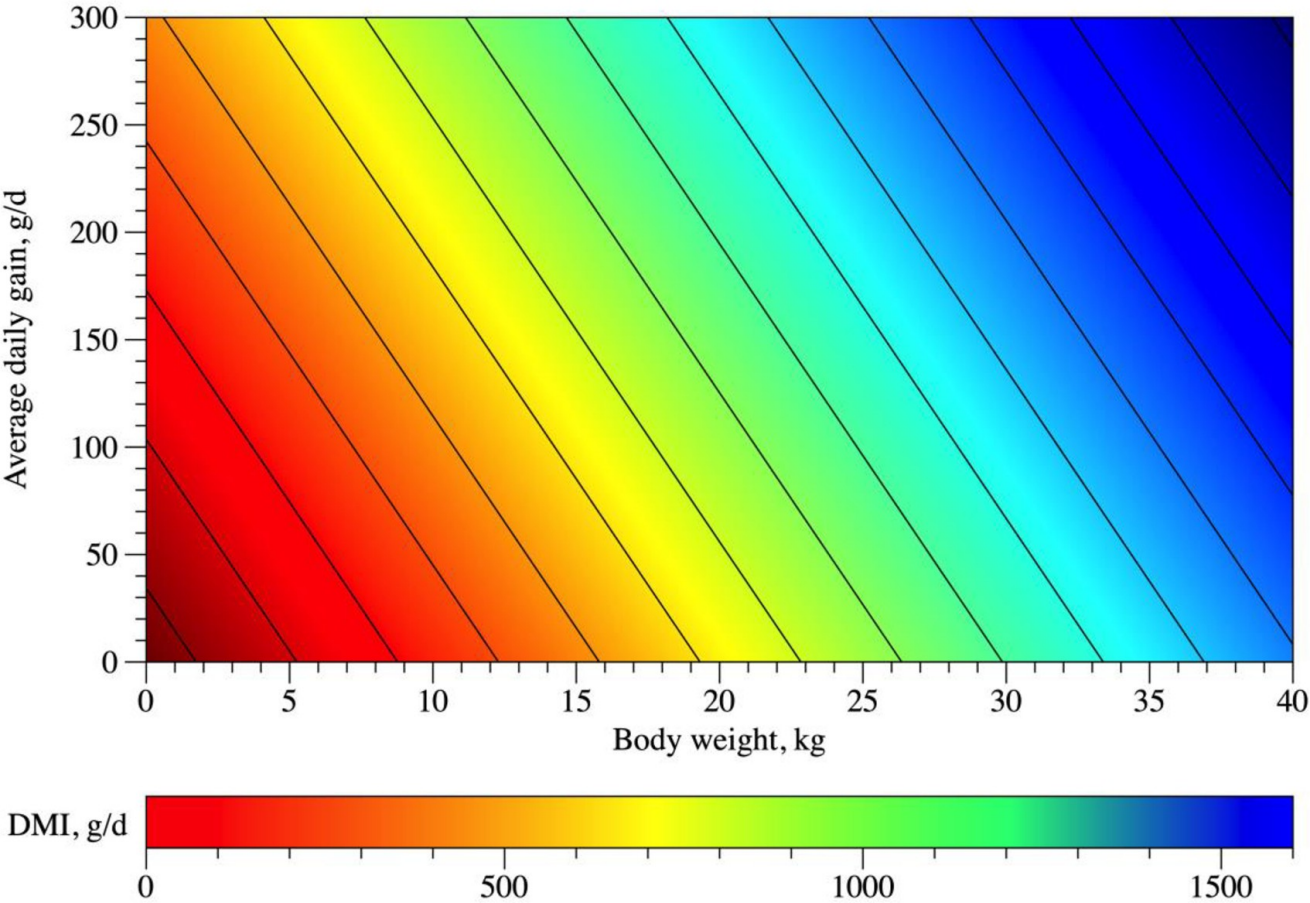
Relationship between Dry Matter Intake (DMI, g/day), Average Daily Gain (ADG, g/day) and Body Weight (BW, kg) in hair sheep raised in tropical regions.

Dry matter intake estimates were expressed in g/day and g/kg BW. The effect of sex on the intercept (P = 0.066), β_1_ (P = 0.745), β_2_ (P = 0.787), β_3_ (P = 0.061) and β_4_ (P = 0.564) from Eq 1 was not significant. Parameters in Eq 2 were also not influenced by sex (P = 0.720 for the intercept, P = 0.868 for β_1_, P = 0.248 for β_2_, P = 0.230 for β_3_ and P = 0.636 for β_4_). DMI prediction models included the variables ADG and BW (Fig 2) as presented in Eqs 8 and 9. The relation between DMI g/kg BW and dietary characteristics Dv and Bv was tested (Eqs 10 and 11). The effect of sex on the intercept (P = 0.543), β_1_ (P = 0.467) and β_2_ (P = 0.425) from Eq 3 was not significant. Similarly, coefficients from Eq 4 were not affected by sex (P = 0.374 for the intercept, P = 0.417 for β_1_ and P = 0.661 for β_2_).

$${DMI}_{(g/day)}=50.5773_{\pm 71.0504}+1.4423_{\pm 0.1704}\times ADG+28.4406_{\pm 2.9697}\times BW$$

$$\left(n=389;\;\sigma_{e}{}^{2}=12295.85;\;r^{2}=0.86;\;\mathrm{AIC}=4731.5;P<0.001\right)$$ (8)

$${DMI}_{\left(g/kgBW\right)}=42.1088_{\pm 4.7298}+0.05516_{\pm 0.009427}\times ADG-0.4402_{\pm 0.1865}\times BW$$

$$\left(n=413;\;\sigma_{e}{}^{2}=11.7586;\;r^{2}=0.81;AIC=2457.1;\;P<0.001\right)$$ (9)

$${DMI}_{(g/kgBW)}={-17.7696}_{\pm 28.2208}+0.1778_{\pm 0.08566}\times Dv-0.00014_{\pm 0.000066}\times {Dv}^{2}$$

$$\left(n=178;\;\sigma_{e}{}^{2}=14.2825;\;r^{2}=0.65;\;AIC=1073.9;P<0.001\right)$$ (10)

$${DMI}_{(g/kgBW)}=42.1810_{\pm 1.7579}-0.01426_{\pm 0.006876}\times Bv$$

$$\left(n=226;\;\sigma_{e}{}^{2}=12.9961;\;r^{2}=0.62;\;AIC=1325.2;\;P<0.001\right)$$ (11)

where DMI = dry matter intake; ADG = average daily gain (g/day); BW = body weight (kg); Dv = D-value (g/kg DM); Bv = B-value (g/kg DM).

**Fig 3:**
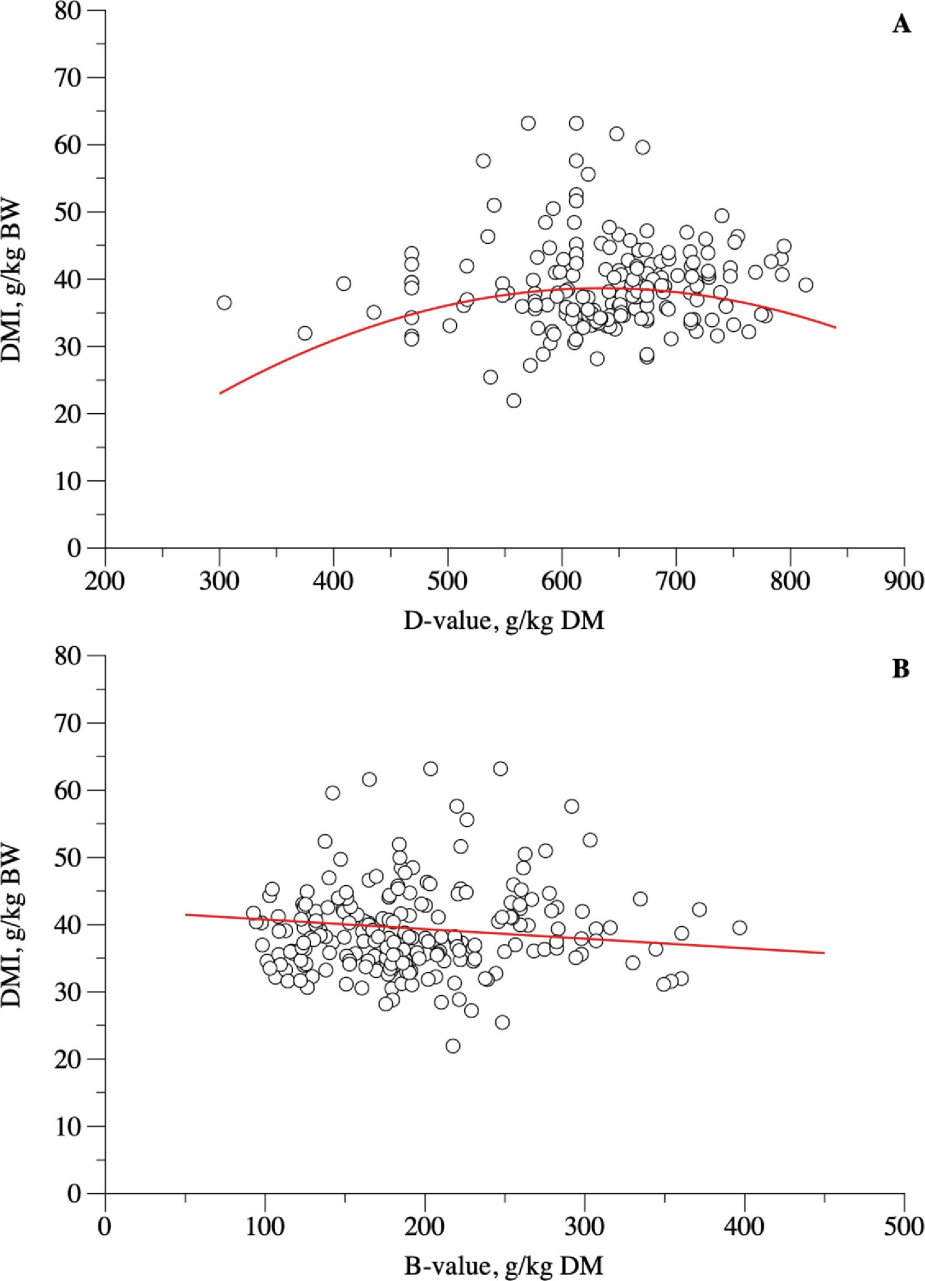
Relationship between dry matter intake (DMI, g/kg BW) and D-value (g/kg DM; A) or B-value (g/kg DM; B) in hair sheep raised in tropical regions.

Considering an ADG of 100 g, the estimated DMI from Eq 8 ranged from 479.21 to 1332.43 for animals weighing 10 to 40 kg, on average (Table 2). Dry matter intake as a function of Dv (Fig 3A, Eq 10) showed a quadratic behavior, whose point of maximum DMI (38.69 g/kg BW) was obtained at 634.1 g/kg DM Dv. On the other hand, DMI decreased linearly as Bv of the diet increased (Fig 3B, Eq 11).

**Table 2:** Prediction of Dry Matter Intake (DMI) of hair sheep raised in tropical regions, and comparison with data from the literature.

ADG^†^	BW*	DMI g/day						
		Eq 8^§^	Eq 9^‡^	NRC (2007)	AFRC (1993)	Cannas et al. (2004)	INRA (2018)	Vieira et al. (2013)
100	10	479.21	432.23	-	-	392.74	-	526.07
200	10	623.44	487.39	-	-	507.08	-	613.07
300	10	767.67	542.54	-	-	611.47	-	700.07
100	20	763.62	776.41	630.00	700.00	650.11	630.00	808.97
200	20	907.85	886.74	830.00	1000.00	749.55	820.00	895.97
300	20	1052.08	997.06	1200.00	-	840.34	1200.00	982.97
100	30	1048.03	1032.56	-	900.00	885.02	-	1091.87
200	30	1192.26	1198.04	1200.00	1400.00	980.49	1040.00	1178.87
300	30	1336.49	1363.52	1250.00	-	1067.66	1240.00	1265.87
100	40	1332.43	1200.67	-	1200.00	1115.08	-	1374.77
200	40	1476.66	1421.31	-	1900.00	1220.89	1250.00	1461.77
300	40	1620.89	1641.95	1290.00	-	1317.51	1450.00	1548.77

^†^ADG = average daily gain.

*BW = body weight.

^§^DMI estimated from Eq 8: ${DMI}_{(g/day)}=50.5773_{\pm 71.0504}+1.4423_{\pm 0.1704}\times ADG+28.4406_{\pm 2.9697}\times BW$

^‡^DMI estimated from Eq 9: ${DMI}_{\left(g/kgBW\right)}=42.1088_{\pm 4.7298}+0.05516_{\pm 0.009427}\times ADG-0.4402_{\pm 0.1865}\times BW$.

**Fig 4:**
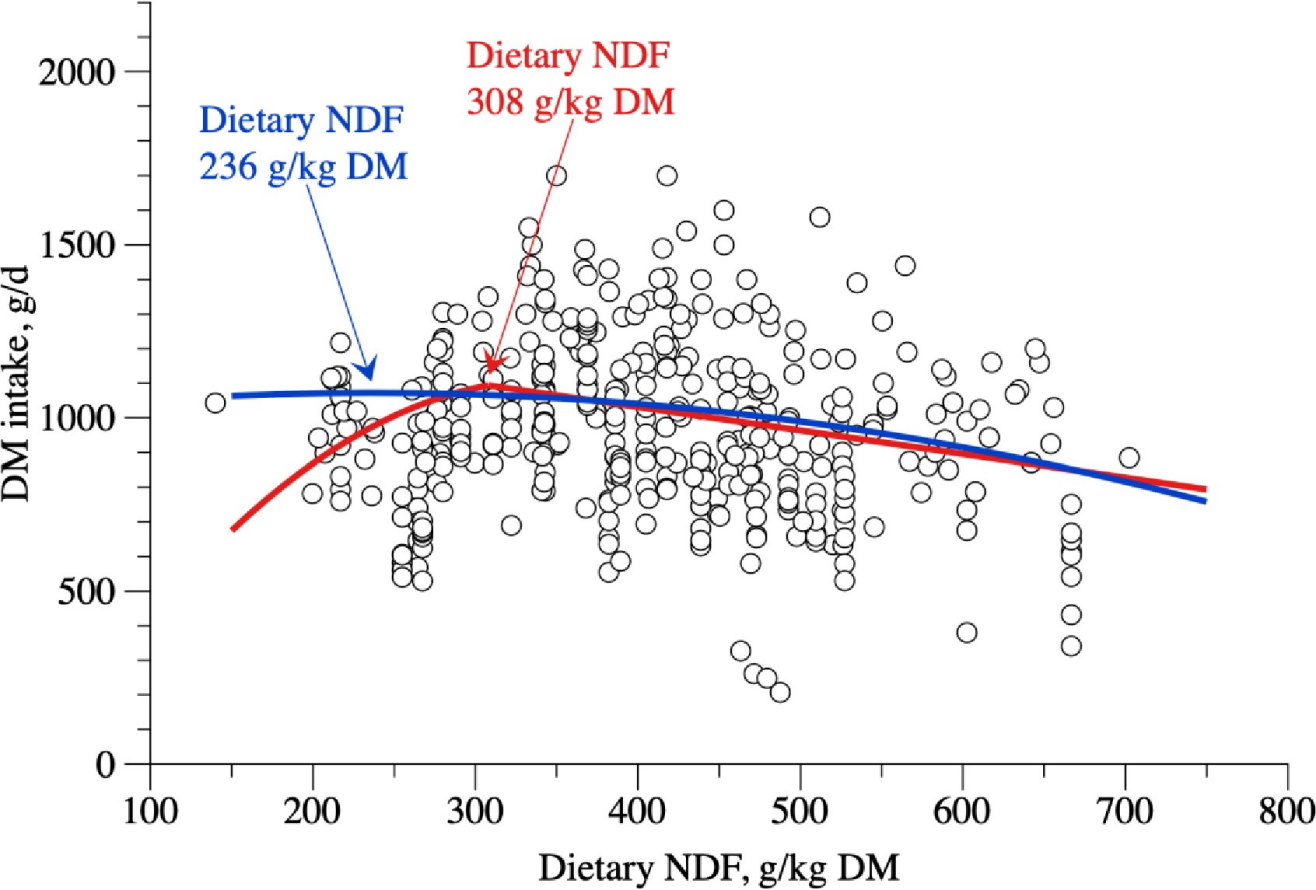
Relationship between dry matter intake (DMI, g/day) and neutral detergent fiber (NDF, g/kg DM) content on diet of hair sheep raised in tropical regions.

Two different approaches were used to evidence the relationship between DMI and NDF contents of the diet (Fig 4). The broken-line method was applied to identify different slopes to peak (Eq 12), as described previously [25], and the single equation quadratic model (Eq 13). The point of maximum DMI of the broken-line and the quadratic models were 1093.95 g/d and 1142.85 g/d at NDF contents of 308 g/kg DM and 236 g/kg DM, respectively.

$$DMI\;(g/d)=\left\{ \begin{array}{c} when\;dietary\;NDF<308\;g/kg={-245.14}_{\pm 376.31}+7.8281_{\pm 2.2616}\times NDF-0.0113_{\pm 0.0034}{NDF}^{2}\\ when\;dietary\;NDF>308\;g/kg=1303.03_{102.58}-0.6787_{0.2065}\times NDF \end{array}\right.$$

$$\left(n=441;\;\sigma_{e}{}^{2}=10551.3;\;r^{2}=0.79;\;AIC=3704.0;\;P<0.020\right)$$ (12)

$$DMI(g/d)=1005.77_{\pm 123.55}+0.5627_{\pm 0.5252}\times NDF-0.0012_{\pm 0.0006}{NDF}^{2}$$

$$\left(n=308;\;\sigma_{e}{}^{2}=11485.4;\;r^{2}=0.79;\;AIC=3967.5;\;P<0.041\right)$$ (13)

### Neutral detergent fiber intake

**Fig 5:**
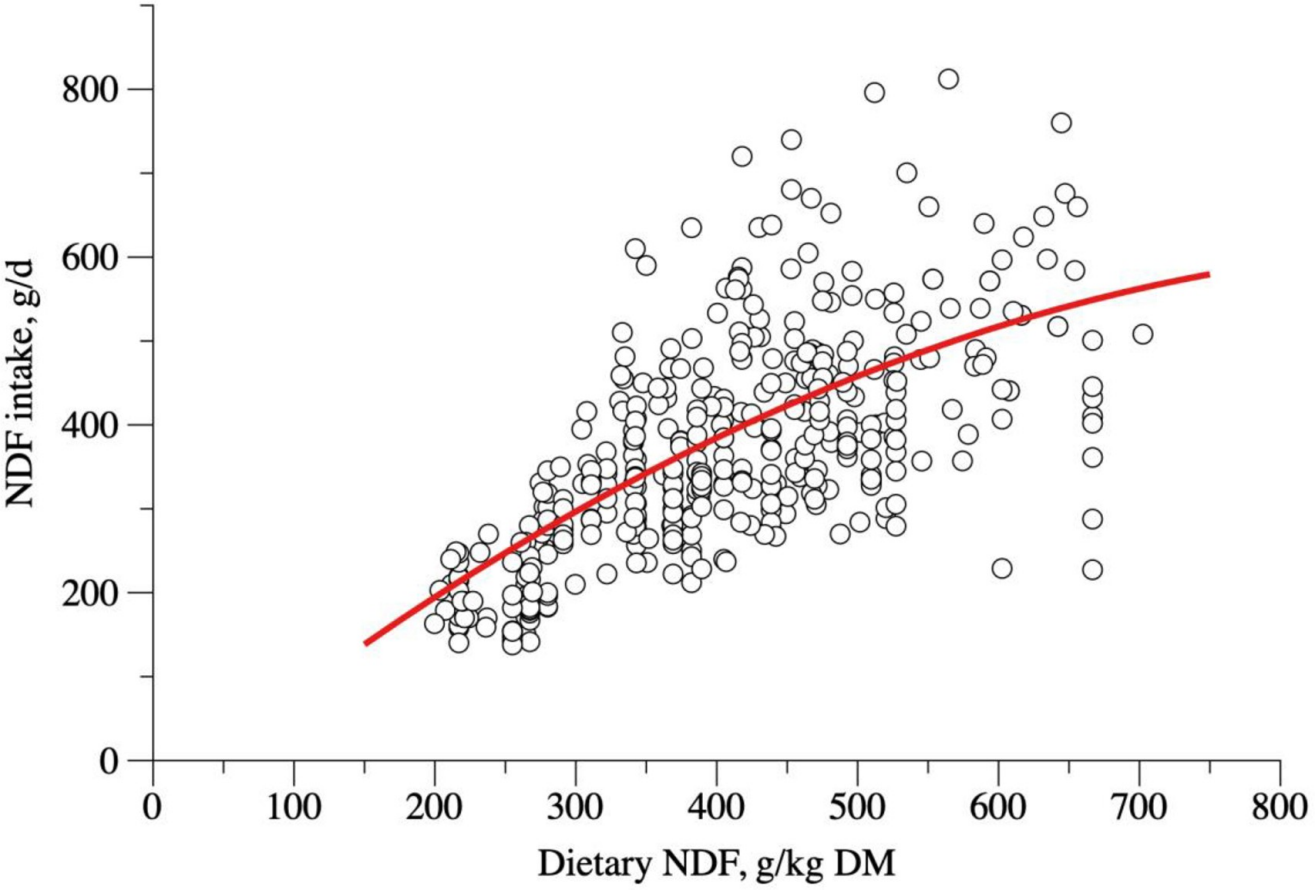
Relationship between neutral detergent fiber intake (NDFI, g/day) and neutral detergent fiber (NDF, g/kg DM) content on diet of hair sheep raised in tropical regions.

**Fig 6:**
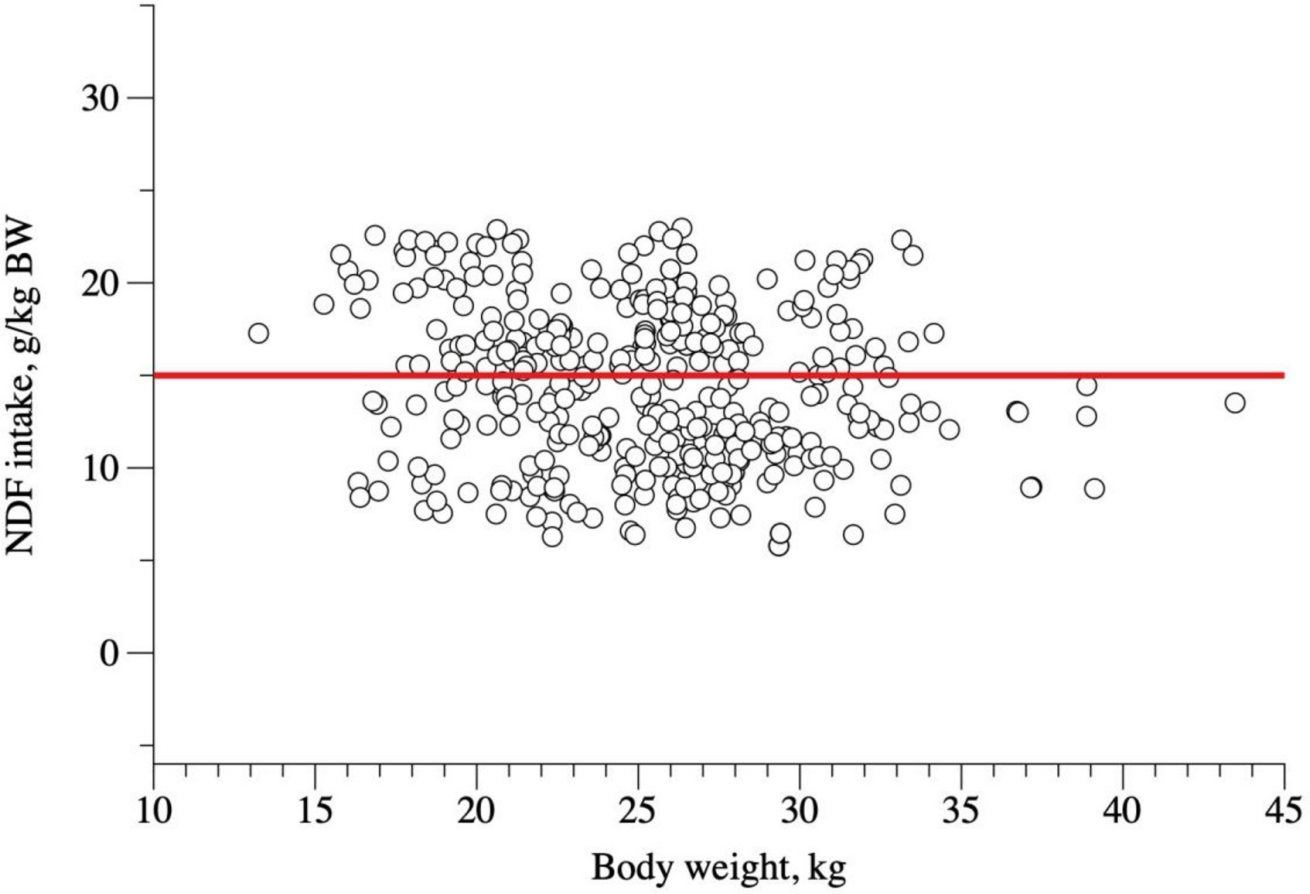
Relationship between neutral detergent fiber intake (NDFI, g/kg BW) and body weight (BW, kg) of hair sheep raised in tropical regions.

The NDFI as a function of dietary NDF content (Fig 5, Eq 14) revealed a quadratic behavior, but it was not possible to determine its maximum point because the point of maximum NDF was out of the data range. Coefficients from Eq 6 were not affected by sex (P = 0.233 for the intercept, P = 0.200 for β_1_ and P = 0.180 for β_2_). We could observe an average NDFI around 15 g/kg BW (Fig 6, Eq 15) in the relationship between NDFI (g/kg BW) and BW. Sex did not influence the intercept (P = 0.137), β_1_ (P = 0.163) or β_2_ (P = 0.212) from Eq 7.

$${NDFI}_{(g/day)}={-52.2187}_{\pm 47.7718}+1.3773_{\pm 0.2292}\times {NDF}_{\left(g/kgDM\right)}-0.0007_{\pm 0.0002}\times {NDF}_{\left(g/kgDM\right)}^{2}$$

$$\left(n=409;\;\sigma_{e}{}^{2}=2343.58;\;r^{2}=0.85;\;AIC=4626.3;\;P<0.001\right)$$ (14)

$${NDFI}_{(g/kgBW)}=149.93(\pm 5.21)$$

$$\left(n=345;\;\sigma_{e}{}^{2}=5.76;\;r^{2}=0.66;\;AIC=1957.6;\;P<0.001\right)$$ (15)

where NDFI = neutral detergent fiber intake; NDF = dietary neutral detergent fiber.

### Model validation

Model predictions were accurate and precise, since most of the MSEPs were represented by random errors, except by Eq 15 (Table 3). Also, the analysis of CCC and its decomposition on Cb exhibited values close to 1 (theoretical perfect fit) indicating that the models presented good accuracy and precision.

**Table 3:** Statistics of adequacy evaluation of the tested models.

Eq^†^	Response Variable	MSEP*	MSEP decomposition, %			CCC^§^	Cb^¶^	*r*
			MB^‡^	Slope	Random			
8	DMI, g/kg BW	11.76	0.00	0.66	99.34	0.91	0.99	0.84
9	DMI, g/d	6480.02	0.00	0.07	99.92	0.93	1.00	0.87
10	DMI, g/kg BW	14.28	0.02	0.42	99.57	0.80	0.97	0.68
11	DMI, g/kg BW	13.00	0.00	0.31	99.69	0.78	0.97	0.65
12	DMI, g/d	10603.27	0.07	0.72	99.21	0.90	0.99	0.82
13	DMI, g/d	11485.37	0.07	0.57	99.37	0.90	0.99	0.82
14	NDFI, g/d	2343.59	0.01	0.05	99.94	0.92	1.00	0.86
15	NDFI, g/kg BW	17672.35	94.55	5.42	0.03	0.01	0.02	0.69

^**†**^Eq = Equation.

*MSEP = mean square error of prediction.

^**‡**^MB = mean bias.

^**§**^CCC = concordance correlation coefficient.

^¶^Cb = bias correction factor.

## Discussion

The sex effect on animal DMI is well studied and it is known that it influences voluntary intake of cattle, and, in certain conditions, sheep and goats [26–28]. In the present study, the effect of sex class (females, and non-castrated or castrated males) on the coefficients of all equations was evaluated, but it was not significant. Indeed, several studies observed similar DMI for the different sex classes of sheep, averaging 15–35 kg BW [29–31]. However, the NRC system considers different nutritional requirements for male and female sheep based on their mature weights, which are not the same. It is important to notice that a limitation of our database could be responsible for the results evidenced herein, since the P-values of sex effects on the coefficients in Eq 8 were close to 0.05. The low BW of the animals (averaging 24.69 kg, below animals’ mature weight) and the small range of the data probably minimized sex class effects. Thus, further studies on sheep raised in tropical areas, especially females and uncastrated males, with average BW higher than 35 kg, are essential to study the effects of sex class on DMI.

Dry matter intake values, estimated from Eq 8, were similar to those resported by a Brazilian study [16] and both were divergent from traditional recommendations for sheep (NRC, AFRC, CNCPS-S and INRA) [13–15, 32]. Our predicted DMI was similar to those of confined Santa Ines lambs raised in Brazil [16] (Table 2, Eq 8), considering a BW of 10–40 kg and an ADG of 100–300 g/day. It is clear that we cannot underestimate the fact that in tropical areas forage usually is poorer in quality than in temperate areas, which probably influenced the results found herein. However, in the present study we did not aimed to separate the influence of climatic condition and forage quality. We would like to know if it is possible to predict DMI and NDFI using the presented variables which could be useful for lambs’ producers (excepting Dv and Bv, which are for scientific proposes, only). In addition, as we stated before, such studies are scarce in tropical areas. Besides, most of the authors did not state the meteorological condictions of their experiments (S2 Table in S1 File). Lastly, we had a high variation in NDF sources, which should represent the variability in those regions. This error, once included in the model will increase power of the model to accurate predict intake for the region, even if it might increase the error in specific conditions or NDF sources. Such results highlight that models from non-tropical regions are not able to predict DMI efficiently and show the importance of studies in this subject. On average, our estimates for the same BW gain were greater than those studies conducted in temperate areas [13–15, 32]. Usually, tropical conditions’ diets have lower fiber quality when compared with diets from temperate regions. Thus, we suspect that diet quality is not playing the major role in these estimates once our DMI estimates would be lower than those of the temperate regions (Table 2). Therefore, differences observed herein might be due to a lower feed efficiency of hair sheep when compared with wool sheep raised in temperate regions. It is worthy noting that if wool sheep were raised in tropical environments, it is likely that hair sheep would present a greater efficiency because of their greater adaptation to heat stress situations [32].

Besides, it is worth noting that NRC [14] and AFRC [13] include parameters other than ADG and BW in their estimates. The AFRC system [13] considers metabolic BW and a correction based on the metabolizable energy content of diet to estimate the DMI of growing sheep. The NRC system [14] uses the standard reference weight at a body score of 2.5 and considers the coefficient of total tract apparent digestibility of diet as 0.80. For growing sheep, they suggest a correction based on the energy content of the diet and the multiple of energy intake above maintenance. Additionally, the NRC [14], includes the variable “weight to maturity” in the prediction model of the DMI. In the present study, the variable weight at maturity was not included in the DMI models because our objective was to estimate the DMI of growing sheep, whose weight variation is relatively small. The CNCPS-S estimates DMI using the full-body weight, but not ADG [33]. All these dissimilarities could justify part of the observed differences when comparing Eq 8 with others.

The INRA system [15] considers dietary characteristics, as it uses the principle of fill unit, which takes in account the intake capacity of the animal and the fill value of feedstuffs. In the present study, dietary characteristics were considered, but in a different methodology from NRC [14] and INRA [15]. Although we used different approaches, the adjustments to represent the relationship between DMI and NDF were unsatisfactory (Fig 4), but the broken-line approach performed better than the quadratic model, because of the lower mean standard error (Fig 3). A similar issue had been observed previously in beef and dairy cattle [8]; nevertheless, a relationship between DMI and dietary characteristics was observed. The concepts of Dv and Bv were applied as an attempt to describe the association between DMI, digestible OM and undigested NDF fractions that would improve DMI estimates when compared to using dietary characteristics.

The potentially digestible fraction of diet (Dv) as well as the undigested fraction of fiber (Bv) are important features of ruminants’ diets, especially in tropical areas, where fiber degradation presents high variation [34, 35]. Both Dv and Bv were associated with DMI (Fig 3A and 3B). Dv represents the dietary apparently digestible OM and presented a quadratic relationship with DMI with a point of maximum intake at a D-value of 634.1 g/kg DM (Eq 10). Bv, on the other hand, was found to decrease linearly with DMI (Eq 11), which was expected, since an increase in Bv represents an increase in bulkiness effect, highlighting its potential to be used as a dietary but also as an animal parameter in modelling. Neutral detergent fiber degradation in the rumen is variable and follows heterogeneous patterns within the same feed and between feeds [34–36]. Besides, other factors, such as particle size, flow of digesta, and potentially digestible fraction of fiber [37] can influence DMI. Despite we found a good fit when estimating DMI from NDF, we recommend using the equation with caution, since NDF content was described previously as unable to accurately predict DMI [8, 37], which could be due to lacking information regarding ruminal signals regulating DMI. Thus, further studies with Dv and Bv would confirm the real needing of these variables in estimating DMI.

For years, researchers have studied metabolic and bulking regulation of DMI as mutually exclusive theories, in which a point of maximum DMI can be found according to the level of dietary fiber. Before this point, energy consumption is greater and metabolic signals regulate DMI [38]. From this point, bulking effects limit DMI, since NDF ferments and passes more slowly through the reticulorumen than the other dietary constituents, leading to a greater filling effect [38]. Thus, such concept does not consider a possible integration of the effects of both metabolic signals and bulking. It seems biologically unlikely that metabolic and physical mechanisms work as a dual phase theory, since energy demand being the major influencer of voluntary intake, it would be logical that changes in energy demand would affect gastrointestinal tract fill capacity [8] and rumen feed passage rate. Because Dv and Bv need more information to be estimated and is a more complex concept, we are not proposing its use for farmers, but equations generated in this study can be useful for scientists to better understand sheep DMI and to demonstrate that feed intake regulation is dependent of an integration between metabolic signals and physical constraints.

A quadratic relationship was observed between dietary NDF and NDFI (Fig 5; Eq 14). When the NDFI was estimated in terms of g/kg BW and its association with BW studied, we observed an average NDFI of around 15 g/kg BW. Thus, NDFI is, on average, 1.5% of the sheep BW (Fig 5). Nevertheless, this model had a low goodness of fit, and this information should be used with care, and future studies should look closely at this subject.

The equations developed herein are applicable primarily to growing and finishing hair sheep raised in tropical conditions in feedlot systems. So, the use of the following equations to estimate DMI and NDFI of hair sheep raised in tropical regions is recommended:

$${DMI}_{(g/day)}=50.5773_{\pm 71.0504}+1.4423_{\pm 0.1704}\times ADG+28.4406_{\pm 2.9697}\times BW;\;{DMI}_{\left(g/kgBW\right)}=42.1088_{\pm 4.7298}+0.05516_{\pm 0.009427}\times ADG-0.4402_{\pm 0.1865}\times BW;\;{NDFI}_{(g/day)}={-52.2187}_{\pm 47.7718}+1.3773_{\pm 0.2292}\times {NDF}_{\left(g/kgDM\right)}-0.0007_{\pm 0.0002}\times {NDF}_{\left(g/kgDM\right)}^{2}.$$

Prediction of DMI of animals under grazing conditions is often complex, given the heterogeneity and variability in the available amount of forage and the selective behavior of the animals, thus further studies on grazing lambs are essential to perform a new meta-analysis aiming to predict DMI and NDFI of lambs under grazing conditions. Due to a variety of reasons, such as the lack of meteorological data, the isolation of climate conditions and forage quality effects were not possible at this moment. Additionally, the low body weight of lambs in our data base and the absence of sex effect in our equations suggests some limitations of our equations. Nevertheless, this meta analysis is markable in studying DMI and NDFI in lambs raised in tropical areas, but further experiments addressing the limitations listed herein are needed to generate more robust equations.

## Conclusions

The preset study was able to fit accurate equations to predict DMI and NDFI to hair sheep raised in tropical conditions. Two equations were fit to predict DMI from BW and ADG. One equation was fit to predict NDFI from NDF content on diet. In addition, it is suggested to consider the value of 1.5% BW to estimate the NDFI (g/kg BW) of hair sheep raised in tropical areas. Using Dv and Bv concepts was satisfactory to describe an integrated mechanism between metabolic and bulking regulation of DMI in sheep.

## Supporting information

S1 File (PDF)

S1 Data (XLSX)
