# Supplementary material for: ‘Meta-analysis of dry matter intake and neutral detergent fiber intake of hair sheep raised in tropical areas’
Source: PLoS One. 2020 Dec 22;15(12):e0244201. doi: 10.1371/journal.pone.0244201 (PMC7755186; doi:10.1371/journal.pone.0244201)
Supplement: S1 File — (PDF) [file pone.0244201.s001.pdf]

Supporting document

Meta-analysis of dry matter intake and neutral detergent fiber intake of hair sheep raised in tropical areas

Alessandra Pinto de Oliveira, Camila Soares Cunha, Elzânia Sales Pereira, Stefano Biffani, Ariosvaldo Nunes de Medeiros, Aderbal Marcos de Azevêdo Silva, Marcos Inácio Marcondes

Supplementary Table 1. List of studies used in the Meta-analysis

Study	Reference
1	Pires, A.J.V., Carvalho Júnior, J.N., Silva, F.F., Veloso, C.M., Souza, A.L., Oliveira, T.N., Santos, C.L., Carvalho, G.G.P., Bernardino, F.S. 2004. Farelo de cacau na alimentação de ovinos. Rev.Ceres. 51:33-43.
2	Oliveira, D.S. Avaliação de dietas formuladas conforme o NRC (2007), com ou sem restrição de nutrientes, para cordeiros terminados em confinamento no semiárido brasileiro. 2017, 110p. Thesis (Animal Science PhD). Universidade Federal do Piauí. Teresina.
3	Oliveira, D.S. Avaliação de dietas formuladas conforme o NRC (2007), com ou sem restrição de nutrientes, para cordeiros terminados em confinamento no semiárido brasileiro. 2017, 110p. Thesis (Animal Science PhD). Universidade Federal do Piauí. Teresina.
4	Fernandes Júnior, F., Ribeiro, E.L.A., Castro, F.A.B., Mizubuti, I.Y., Silva, L.D.F., Pereira, E.S., Pinto, A.P., Barbosa, M.A.A.F., Koritiaki, N.A. 2015. Desempenho, consumo e morfometria in vivo de cordeiros Santa Inês alimentados com rações contendo torta de girassol em substituição ao farelo de algodão. Arq. Bras. Med. Vet. Zootec. 67:438-491. DOI: 10.1590/1678-7071
5	Valença, R.L., Ferreira, A.C.D., Santos, A.C.P., Silva, B.C.D., Santos, G.R.A., Lima, J.U.N., Pereira, M.A., 2017. Silagem de bagaço de laranja na alimentação de cordeiros: consumo de nutrientes, desempenho e avaliação econômica. Arch. Zootec. 66:81-87. DOI: 10.21071/az.v66i253.2129
6	Grandis, F.A., Ribeiro, E.L.A., Mizubuti, I.Y., Silva, L.D.F., Bumbieris Júnior,V.H., Prado, O.P.P., Constantino, C., Fernandes Júnior, F., Mangilli, L.G., Pereira, E.S. Desempenho, consumo de nutrientes e comportamento ingestivo de cordeiros alimentados com diferentes teores de torta de soja em substituição ao farelo de soja. 2015. Rev. Bras. Saúde Prod. Anim. 16:558-570. DOI: 10.1590/S1519-99402015000300008

7	Azevedo, J.A.G., Souza, L.L., Salt, M.P.F., Nascimento, L.S., Almeida, F.M., Pereira, L.G.R., Silva, R.R., Oliveira, G.A., Ferreira, D.M.F., Almeida, V.V.S., 2015. Substituição do milho pela silagem de jaca em dietas para cordeiros confinados. Semina: Ciênc. Agrar. 36:1599-1608. doi: 10.5433/1679-0359.2015v36n3p1599
8	Andrade, J.O. Silagem da ponta de cana-de-açúcar aditivada com resíduo de cervejaria desidratado na alimentação de ovinos. 2013, 58p. Thesis (Animal Science PhD). Universidade Federal de Viçosa, Viçosa.
9	Rufino, L.D.A. Substituição do farelo de soja por levedura seca inativa em dieta de ovinos. 2011, 51p. Thesis (Animal Science Masters). Universidade Federal de Viçosa, Viçosa.
10	Oliveira, H.C. Farelo de mamona detoxificado na dieta de cordeiros. 2013, 71p. Thesis (Animal Science PhD). Universidade Federal de Viçosa. Viçosa.
11	Nogueira, A.S. Torta de licuri na alimentação de ovinos. 2013, 89p. Thesis (Animal Science PhD). Universidade Federal de Viçosa, Viçosa.
12	Nicory, I.M.C., Carvalho, G.G.P., Ribeiro, O.L., Santos, S.A., Silva, F.F., Silva, R.R., Lopes, L.S.C., Souza, F.N.C., Freitas Júnior, J.E., 2015. Productive and metabolic parameters in lambs fed diets with castor seed meal. Livest. Sci. 181:171-178. DOI:10.1016/j.livsci.2015.09.015
13	Santos, V.C., Ezequiel, J.M.B., Morgado, E.S., Fávaro, V.R., D'Áurea, A.P., Sousa Júnior, S.C. 2014. Desempenho e digestibilidade de componentes nutritivos de dietas contendo subprodutos de oleaginosas na alimentação de cordeiros. Semina: Ciênc. Agrár. 35:1577-1586. DOI: 10.5433/1679-0359.2014v35n3p1577
14	Perez, H.L. Milho, amido ou caroço de algodão associados a glicerina bruta em dietas para ovinos. 2015, 87p. Thesis (Animal Science PhD). Universidade Estadual Paulista, Jaboticabal.
15	Cordão, M.A.C, Bakke, O.A., Pereira, G.M., Silva, A.M.A., Pereira Filho, J.M., Vitorino, P.V.V., Silva, A.G.P.F., Moura, A.V.C., 2016. Jurema preta (*Mimosa tenuiflora* (Willd. Poiret) na dieta de cordeiros. Agrarian. 9:287-295.
16	Camurça, D.A., Neiva, J.N.M., Pimentel, J.C.M., Vasconcelos, V.R., Lôbo, R.N.B., 2002. Desempenho Produtivo de ovinos alimentados com dietas à base de feno de gramíneas tropicais. R. Bras. Zootec. 31: 2113-2122.
17	Lima, C.A.C., Lima, G.F.C., Costa, R.G., Medeiros, A.N., Aguiar, E.M., Lima Júnior, V., 2012. Efeito de níveis de melão em substituição ao milho moído sobre o desempenho, o consumo e a digestibilidade dos nutrientes em ovinos Morada Nova. Rev. Bras. Zootec. 41:164-171. DOI: 10.1590/S1516-35982012000100024
18	Lima, C.A.C., Lima, G.F.C., Costa, R.G., Medeiros, A.N., Aguiar, E.M., Lima Júnior, V., 2012. Efeito de níveis de melão em substituição ao milho moído sobre o desempenho, o consumo e a digestibilidade dos nutrientes em ovinos Morada Nova. Rev. Bras. Zootec. 41:164-171. DOI: 10.1590/S1516-35982012000100024
19	Castro, J.M.C, Silva, D.S., Medeiros, A.N.M., Pimenta Filho, E.C., 2007. Desempenho de cordeiros Santa Inês alimentados com dietas completas contendo feno de maniçoba. R. Bras. Zootec. 36:674-680.

20	Amorim, R.N.L. Consumo, digestibilidade, desempenho e características da carcaça de ovinos alimentados com resíduo do maracujá (*Passiflora edulis* L.) em substituição ao milho. 2015, 56p. Thesis (Animal Science Masters). Universidade Federal Rural do semi-árido. Mossoró.
21	Medeiros, G.R. Efeito dos níveis de concentrado sobre o desempenho, características de carcaça e componentes não carcaça de ovinos morada nova em confinamento. 2006, 109p. Thesis (Animal Science PhD). Universidade Federal Rural de Pernambuco. Recife.
22	Santana, E.O.C. Desempenho e comportamento ingestivo de ovinos alimentados sem volumoso. 2015, 96p. Thesis (Animal Science PhD). Universidade Federal do Sudoeste da Bahia. Itapetinga.
23	Silva, J.L., Ribeiro, K.G., Pereira, O.G., Valadares Filho, S.C., Pina, D.S., Paulino, P.V.R., 2015. Performance and carcass characteristics of Santa Inês lambs fed protein diets. Rev. Ciênc. Agrár. 38: 49-57. DOI: 10.1590/S1516-35982011001200039
24	Cardoso, D.B., Carvalho, F.F.R., Medeiros, G.R., Guim, A., Cabral, A.M.D., Véras, R.M.L., Santos, K.C., Dantas, L.C.N., Nascimento, A.G.O., 2019. Levels of inclusion of spineless cactus (*Nopalea cocheillifera* Salm Dyck) in the diet of lambs. Anim. Feed Sci. Technol. 247:23-31. DOI: 10.1016/j.anifeedsci.2018.10.016
25	Oliveira, A.P.D., Bagaldo, A.R., Loures, D.R.S., Bezerra, L.R., Moraes, S.A., Yamamoto, S.M., Araújo, F.I., Cirne, L.G., Oliveira, R.L., 2018. Effect of ensiling gliricidia with cassava on silage quality, growth performance, digestibility, ingestive behavior and carcass traits in lambs. Anim. Feed Sci. Technol. 241:198-209. DOI: 10.1016/j.anifeedsci.2018.05.004
26	Lage, J.F., Paulino, P.V.R., Pereira, L.G.R., Valadares Filho, S.C., Oliveira, A.S., Detmann, E., Souza, N.K.P., Lima, J.C.M., 2010. Glicerina bruta na dieta de cordeiros terminados em confinamento. Pesq. Agropec. Bras. 45:1012-1020.
27	Mattos, C.W. Associação de palma forrageira (*Opuntia ficus-indica* Mill) e feno de erva-sal (*Atriplex nummularia* L) em dietas para cordeiros santa inês em confinamento. 2009, 101p. Thesis (Animal Science PhD). Universidade Federal Rural de Pernambuco. Recife.
28	Mendes, C.Q., Susin, I., Pires, A.V., Nussio, L.G. Araújo, R.C., Ribeiro, M.F., 2008. Desempenho, parâmetros da carcaça e comportamento ingestivo de cordeiros alimentados com cana-de-açúcar ensilada ou in natura. Arq. Bras. Med. Vet. Zootec. 60:733-740.
29	Pereira, M.S., Ribeiro, E.L.A., Mizubuti, I.Y., Rocha, M.A., Kuraoka, J.T., Nakaghi, E.Y.O., 2008. Consumo de nutrientes e desempenho de cordeiros em confinamento alimentados com dietas com polpa cítrica úmida prensada em substituição à silagem de milho. Rev. Bras. Zootec. 37:134-139. DOI: 10.1590/S1516-35982008000100020
30	Silva, H.G.O., Pires, A.J.V., Carvalho, G.G.P., Veloso, C.M., Silva, F.F. 2008. Capim-elefante amonizado e farelo de cacau ou torta de dendê em dietas para ovinos em crescimento. Rev. Bras. Zootec. 37:734-742. DOI: 10.1590/S1516-35982008000400021
31	Gastaldello Júnior, A.L., Pires, A.V., Susin, I., Mendes, C.Q., Ferreira, E.M., Mourão, G.B., 2010. Desempenho e características de carcaça de cordeiros alimentados com dietas contendo alta proporção de concentrado adicionadas de agentes tamponantes. Rev. Bras. Zootec. 39:556-562.

32	Cunha, M.G.G., Carvalho, F.F.R., Véras, A.S.C., Batista, A.M.V., 2008. Desempenho e digestibilidade aparente em ovinos confinados alimentados com dietas contendo níveis crescentes de caroço de algodão integral. Rev. Bras. Zootec. 37:1103-1111. DOI: 10.1590/S1516-35982008000600022
33	Coelho, C. P. Desempenho de ovinos da raça santa inês alimentados com silagens com diferentes concentrações de tanino. 2007, 50p. Thesis (Animal Science Masters). Universidade Federal do Sudoeste da Bahia. Itapetinga.
34	Alves, K.S., Carvalho, F.F.R., Véras, A.S.C., Costa, R.G., Santos, E.P., Freitas, C.R.G., Santos Júnior, C.M., Andrade, D.K.B., 2003. Níveis de Energia em Dietas para Ovinos Santa Inês: Digestibilidade Aparente. Rev. Bras. Zootec.32:1962-1968. DOI: 10.1590/S1516-35982003000800021
35	Barreto, C.M., Azevedo, A.R., Sales, R.O., Arruda, F.A.V., Alves, A.A., 2004. Desempenho de Ovinos em Terminação Alimentados com Dietas Contendo Diferentes Níveis de Dejetos de Suínos. Rev. Bras. Zootec. 33:1858-1865. DOI: 10.1590/S1516-35982004000700025
36	Reis, V.A.A., Furusho-Garcia, I.R., Pérez, J.R. 2014. Increasing levels of lipids, using sunflower meal in the diet of finishing lambs on meat quality. 60th International Congress of Meat Science and Technology. Punta del Este, Uruguai.
37	Oliveira, A.O. Requerimentos nutricionais de cordeiros santa inês com peso corporal de 14 a 28 kg. 2012. Thesis (Animal Science Masters). Universidade Federal do Ceará. Fortaleza.
38	Regadas Filho, J.G. Exigências energéticas e proteicas de ovinos Santa Inês em crescimento. 2009, 80p. Thesis (Animal Science Masters). Universidade Federal do Ceará. Fortaleza.
39	Pereira, G.M., 2011. Energy and Protein Requirements of Santa Ines Rams in the Brazilian Semiarid Region. Thesis (Animal Science Masters). Universidade Federal de Campina Grande. Patos.
40	Costa, M.R.G.F., Exigências nutricionais de cordeiros deslanados e validação do modelo Small Ruminant Nutrition System (SRNS). 2012, 113p. Thesis (Animal Science PhD). Universidade Federal do Ceará. Fortaleza.
41	Fontenele, R.M. Exigências nutricionais de cordeiros da raça Somalis brasileira. 2014, 106p. Thesis (Animal Science PhD). Universidade Federal do Ceará. Fortaleza.
42	Pereira, M.W.F. Exigências de energia e proteína para mantença e ganho em ovinos morada nova de diferentes classes sexuais em confinamento. 2017, 70p. Thesis (Animal Science PhD). Universidade Federal do Ceará. Fortaleza.
43	Lima, F.W.R. Exigências nutricionais de cordeiros santa inês castrados e não castrados. 2017, 79p. Thesis (Animal Science PhD). Universidade Federal do Ceará. Fortaleza.
44	Dantas Filho, L.A., Lopes, J.B., Vasconcelos, V.R., Oliveira, M.E., Alves, A.A., Araújo, D.L.C., Conceição, W.L.F., 2007. Inclusão de polpa de caju desidratada na alimentação de ovinos: desempenho, digestibilidade e balanço de nitrogênio. Rev. Bras. Zootec. 36:147-154. DOI: 10.1590/S1516-35982007000100018

45	Costa, R.G., Pinto, T. F., Medeiros, G. R., Medeiros, A. N. , Queiroga, R.C. R. E., Treviño, I. H., 2012. Meat quality of Santa Inês sheep raised in confinement with diet containing cactus pear replacing corn. Rev. Bras. Zootec. 41: 432-437. DOI: 10.1590/S1516-35982012000200028
46	Carvalho, G.G.P., Rebouças, R.A., Campos, F.S., Santos, E.M., Araújo, G.G.L., Gois, G.C., Oliveira, J.S., Oliveira, R.I., Rufino, L.M.A., Azevedo, J.A.G., Cirne, L.G.A., 2017. Intake, digestibility, performance, and feeding behavior of lambs fed diets containing silages of different tropical forage species. Anim. Feed Sci. Technol. 228:140-148. DOI: 10.1016/j.anifeedsci.2017.04.006
47	Oliveira, R.P., Perez, J.R.O., Muniz, J.A., Evangelista, A.R., Souza, J.C., Barcelos, A.F., 2009. Effect of concentrate : voluminous ratio on the performance of santa inês lambs. Ciênc. Agrotec. 33:1637-1647. DOI: 10.1590/S1413-70542009000600025
48	Macome, F., Ronaldo Lopes O, Adriana Regina B, Gherman Garcia Leal A, Larissa Pires B, Mauricio Costa Alves Da S. 2011. Productive performance and carcass characteristics of lambs fed diets containing different levels of palm kernel cake. Rev.MVZ Córdoba, 16:2659-2667. DOI: 10.21897/rmvz.266
49	Aguiar, S.R., Ferreira, M.A., Batista, A.M.V., Carvalho, F.F.R., Bispo, S.V., Monteiro, P.B.S., 2007. Desempenho de ovinos em confinamento alimentados com níveis crescentes de levedura e uréia. Acta Sci. Anim. Sci. 29:411-416.
50	Salvador, F.M. Desempenho e digestibilidade em ovinos da raça santa inês alimentados em diferentes condições de balanços de proteína degradável no rúmen e proteína metabolizável. 2007, 135p. Thesis (Animal Science PhD). Universidade Federal de Lavras. Lavras.
51	Barros e Silva, T.M. Desempenho e características de carcaça de ovinos alimentados com silagem de capim búffel em substituição a silagem de milho. 2014, 76p. Thesis (Animal Science Masters). Universidade Federal do Vale do São Francisco. Petrolina.
52	Araújo, G.G.L., Bade, P.L., Menezes, D.R., Socorro, E.P., Sá, J.L., Oliveira, G.J.C., 2009. Substituição da raspa de mandioca por farelo de palma forrageira na dieta de ovinos.Rev. Bras. Saúde Prod. Anim. 10:448-459.
53	Murta, R.M., Chaves, M.A., Pires, A.J.V., Veloso, C.M., Silva, F.F., Rocha Neto, A.L., Eustáquio Filho, A.E., Santos, P.E.F.S., 2010. Desempenho e digestibilidade aparente dos nutrientes em ovinos alimentados com dietas contendo bagaço de cana-de-açúcar tratado com óxido de cálcio. Rev. Bras. Zootec. 40:1325-1332.
55	Nascimento Júnior, J.R.S. Palma forrageira e resíduo de feijão na alimentação de ovinos em confinamento. 2014, 71p. Thesis (Animal Science Masters). Universidade Federal Rural de Pernambuco. Garanhus.
56	Barros, M.C.C., Marques, J.A., Silva, F.F., Silva, R.R., Guimarães, G.S., Silva, L.L., Araújo, F.L., 2015. Glicerina bruta na dieta de ovinos confinados: consumo, digestibilidade, desempenho, medidas morfométricas da carcaça e características da carne. Semina: Ciênc. Agrár. 36:453-466. DOI: 10.5433/1679-0359.2015v36n1p453
57	Santos, T. R., 2011. Castor Meal Treated with Calcium Oxide, Wet Or Dry Provided for Lambs.Thesis (Animal Science Masters). Universidade Federal de Viçosa, Viçosa.

58	Rodrigues, R. T. S., 2013. Performance and Energy and Protein Requirements of Lambs without Defined Racial Patterns of Different Sexual Classes. Dissertation. Universidade Federal do Vale do São Francisco, Petrolina.
59	Caetano, G.G.G.P. Bagaço de cana de açúcar in natura em substituição parcial à silagem de milho na dieta de ovinos. 2014, 54p. Thesis (Animal Science Masters). Universidade Federal do Mato Grosso. Cuiabá.
60	Carvalho, G.G.P., Pires, A.J.V., Veloso, C.M., Silva, F.F., Silva, R.R., 2006. Desempenho e digestibilidade de ovinos alimentados com farelo de cacau (*Theobroma cacao* L.) em diferentes níveis de substituição. Ciên. Anim. Bras. 7:115-122.
61	Caldas, A.C.A. Desempenho e características de carcaça de ovinos recebendo dietas com feno de jurema preta (*Mimosa tenuiflora*(Wild.) (Poir) como fonte de tanino. 2018, 78p. Thesis (Animal Science Masters). Universidade Federal de Campina Grande, Patos.

S2 Table - Meteorological information about each study used in the meta-analysis.

Study	City	State	Köppen's climate classification
45	São João do Cariri	Paraíba	Dry Semi-arid with low latitude and altitude
46	Petrolina	Pernambuco	Dry Semi-arid with low latitude and altitude
51	Petrolina	Pernambuco	Dry Semi-arid with low latitude and altitude
52	Petrolina	Pernambuco	Dry Semi-arid with low latitude and altitude
25	Petrolina	Pernambuco	Dry Semi-arid with low latitude and altitude
27	Petrolina	Pernambuco	Dry Semi-arid with low latitude and altitude
55	Garanhuns	Pernambuco	Hot tropical sub humid
9	Viçosa	Minas Gerais	Humid subtropical with dry winter and hot summer
23	Viçosa	Minas Gerais	Humid subtropical with dry winter and hot summer
26	Viçosa	Minas Gerais	Humid subtropical with dry winter and hot summer
36	Lavras	Minas Gerais	Humid subtropical with dry winter and hot summer
47	Lavras	Minas Gerais	Humid subtropical with dry winter and hot summer
50	Lavras	Minas Gerais	Humid subtropical with dry winter and hot summer
57	Viçosa	Minas Gerais	Humid subtropical with dry winter and hot summer
59	Santo Antônio de Leverger	Mato Grosso	Humid subtropical with dry winter and hot summer
17	Pedro Avelino	Rio Grande do Norte	Semi-arid
18	Pedro Avelino	Rio Grande do Norte	Semi-arid
19	São João do Cariri	Paraíba	Semi-arid
32	Soledade	Paraíba	Semi-arid
60	Londrina	Paraná	Subtropical without dry season
4	Londrina	Paraná	Subtropical without dry season
6	Londrina	Paraná	Subtropical without dry season

28	Piracicaba	São Paulo	Subtropical without dry season
29	Londrina	Paraná	Subtropical without dry season
31	Piracicaba	São Paulo	Subtropical without dry season
21	Recife	Pernambuco	Tropical monsoon
34	Recife	Pernambuco	Tropical monsoon
49	Recife	Pernambuco	Tropical monsoon
1	Itapetinga	Bahia	Tropical with dry winter
2	Sobral	Ceará	Tropical with dry winter
3	Sobral	Ceará	Tropical with dry winter
5	São Cristóvão	Sergipe	Tropical with dry winter
7	Ilhéus	Bahia	Tropical with dry winter
10	Brasília	Distrito Federal	Tropical with dry winter
11	Ilhéus	Bahia	Tropical with dry winter
13	Goiânia	Goiás	Tropical with dry winter
14	Jaboticabal	São Paulo	Tropical with dry winter
15	Patos	Paraíba	Tropical with dry winter
16	Fortaleza	Ceará	Tropical with dry winter
20	Fortaleza	Ceará	Tropical with dry winter
22	Jequié	Bahia	Tropical with dry winter
24	Campina Grande	Paraíba	Tropical with dry winter
30	Itapetinga	Bahia	Tropical with dry winter
33	Itapetinga	Bahia	Tropical with dry winter
35	Fortaleza	Ceará	Tropical with dry winter
37	Fortaleza	Ceará	Tropical with dry winter
38	Fortaleza	Ceará	Tropical with dry winter
39	Fortaleza	Ceará	Tropical with dry winter
40	Fortaleza	Ceará	Tropical with dry winter
41	Fortaleza	Ceará	Tropical with dry winter
42	Fortaleza	Ceará	Tropical with dry winter
43	Fortaleza	Ceará	Tropical with dry winter
44	Teresina	Piauí	Tropical with dry winter
53	Salinas	Minas Gerais	Tropical with dry winter
56	Itapetinga	Bahia	Tropical with dry winter
58	Fortaleza	Ceará	Tropical with dry winter
61	Patos	Paraíba	Tropical with dry winter
48	Salvador	Bahia	Tropical without dry season
12	São Gonçalo dos Campos	Bahia	Tropical without dry season
8	Cruz das Almas	Bahia	Tropical without dry season
